# The Potential Role of the Leucocyte Immunoglobulin-Like Receptors in Kidney Transplant Rejection: A Mini Review

**DOI:** 10.3389/ti.2024.12995

**Published:** 2024-07-01

**Authors:** Jovanne Palvair, Imane Farhat, Mélanie Chaintreuil, Ludivine Dal Zuffo, Lennie Messager, Claire Tinel, Baptiste Lamarthée

**Affiliations:** ^1^ Université de Franche-Comté, EFS, INSERM, UMR RIGHT, Besançon, France; ^2^ Centre Hospitalier Universitaire de Dijon, Service de Néphrologie et Transplantation Rénale, Université de Bourgogne, Dijon, France

**Keywords:** kidney transplant, allograft rejection, monocyte, LILRs, innate immunity

## Abstract

Antibody-mediated rejection (ABMR) remains one of the main causes of long-term graft failure after kidney transplantation, despite the development of powerful immunosuppressive therapy. A detailed understanding of the complex interaction between recipient-derived immune cells and the allograft is therefore essential. Until recently, ABMR mechanisms were thought to be solely caused by adaptive immunity, namely, by anti-human leucocyte antigen (HLA) donor-specific antibody. However recent reports support other and/or additive mechanisms, designating monocytes/macrophages as innate immune contributors of ABMR histological lesions. In particular, in mouse models of experimental allograft rejection, monocytes/macrophages are readily able to discriminate non-self via paired immunoglobulin receptors (PIRs) and thus accelerate rejection. The human orthologs of PIRs are leukocyte immunoglobulin-like receptors (LILRs). Among those, LILRB3 has recently been reported as a potential binder of HLA class I molecules, shedding new light on LILRB3 potential as a myeloid mediator of allograft rejection. In this issue, we review the current data on the role of LILRB3 and discuss the potential mechanisms of its biological functions.

## Introduction

Two major types of rejection are classically described after kidney transplantation: T cell-mediated rejection (TCMR) and antibody-mediated rejection (ABMR), named after their respective presumed mechanism of injury: T lymphocytes and anti-human leucocyte antigen (HLA) donor-specific antibody (DSA). However, with regard to ABMR, current antibody-targeting treatments are proving to be disappointing, highlighting a lack of a comprehensive understanding of all the immune mechanisms involved [1]. Furthermore, some rejections show both typical and concomitant lesions of ABMR and TCMR and are sometimes referred to as “Mixed rejection,” suggesting that potentially common cellular mechanism are at play in these cases. New advances indicate that allograft lesions can be induced by myeloid cells, independently of DSA. A high heterogeneity in the graft infiltrating cells has been demonstrated, with myeloid cells accounting for up to 80% of those upon rejection [2]. Importantly, murine models of solid organ transplantation suggest that monocyte/macrophage may recognize non-self determinant [3] independently of adaptive immunity [4, 5]. The specific depletion of monocyte/macrophages may additionally preserve the allograft from rejection lesions [6–8]. Recently in mice, an elegant report has identified receptors on the surface of recipient-derived monocytes/macrophages capable of interacting specifically with donor cells via their major histocompatibility complexes and promoting rejection [9]. The authors of this study showed previously that initiation of the primary alloresponse of recipient’s monocytes requires the interaction of their CD47 ligand with SIRP-α [5]. This initiation leads to paired immunoglobulin-like receptors (PIRs) modulation at monocytes membrane and notably PIR-A was identified as responsible of donor’s MHC-I recognition. Deletion of these receptors and/or inhibition of their interaction with the complexes improved long-term allograft survival. Inversely, deletion of PIR-B lead to rapid rejection, underlying the opposite mechanism of both receptors and the balance existing between them. Deletion of one precipitates/accelerates/determine the fate of the grafted organ. They validated their findings in two solid organ transplantation murine models, in both heart and kidney allograft. Importantly, as long as PIRs were expressed on the surface of monocytes, an innate memory response against non-self MHC persisted [9]. These so-called PIRs are the murine orthologs of the human leukocyte immunoglobulin-like receptors (LILRs). In humans, half of these LILRs were described as ligand for class I HLA and could thus play a role in myeloid responses in an allogeneic context [10–13]. The present review aims to understand the involvement of these receptors, with a special focus on LILRB3, in recognizing donor tissue as non-self and activating the recipient’s myeloid cells against the transplanted organ.

### LILRs as an Immunomodulatory Family

LILRs are a family of immunomodulatory receptors expressed on myeloid and lymphoid cell lines [14]. These receptors are structurally similar to killer-cell immunolobulin-like receptors (KIRs), whose role in allograft rejection has been highlighted over the past decade [15]. As for KIRs, their encoding genes are located on chromosome 19q [16], and clustered in centromeric and telomeric regions with opposite transcription directions. Depending on the haplotype, *LILRA6* and *LILRB3* genes have different copy numbers [17, 18]. Within the LILRs’ family, LILRAs and LILRBs subtypes were further defined according to their activators (LILRAs) or inhibitors (LILRBs) functions, the distinction being made according to the activating or inhibitory motifs of their intracellular domain [10]. Both LILRAs and LILRBs are characterized by an extracellular structure comprising several immunoglobulin (Ig) domains. The LILRA-dependent signaling pathways are activated by the recruitment of Src and Syk kinases following the phosphorylation of immunoregulatory tyrosin-based activator motifs (ITAM) sequences present in the intracellular compartment. The Syk and Src kinases phosphorylate PI3K and PLC *γ*2, leading to activation of the PI3K/Akt, NFAT, Ras/ERK, NF-κB, JNK and MAPK pathways. On the opposite, LILRBs signal transduction include a cytoplasmic domain rich in immunoregulatory tyrosin-based inhibitory motifs (ITIM). After binding of the LILRBs ligands, the phosphorylation of the intracellular ITIM sequences induces the recruitment of the Src homology domain phosphatases SHP-1 or SHP-2. Activation of these phosphatases leads to dephosphorylation of ITAM sequences, particularly those carried by neighboring LILRAs, as well as kinases involved in their downstream signaling. Thereby, LILRB ITIM sequences can interfere with effector functions, cytokine secretion as well as immune cell maturation [19]. For instance, activation of LILRB3 was recently reported to induce NF-κB pathway in cancer cells [20].

### LILRB3, a Potential Myeloid Immune Checkpoint

LILRB3, also know n as ILT5 (immunoglobulin-like transcript 5), is emerging as a key player in the modulation of the immune response and has attracted growing interest in biomedical research. In the tumor microenvironment, the surface expression of LILRB3 on myeloid cells was recently correlated to a poor patient survival [21, 22]. In addition, a remarkable work reported LILRB3 binding capacity to class I HLA [23], suggesting a new role in regulating immune cells interaction, and not only in regulating immune cell functions. Recently using single-cell RNA sequencing, we showed that *LILRB3* expression was increased in kidney allograft infiltrating monocytes during ABMR, suggesting LILRB3 involvement in monocyte activation during rejection [24, 25]. Interestingly the high polymorphism of this gene [26] leads to the translation of slightly different molecules. In 2009, Pfistershammer and colleagues reported two major variants of the LILRB3 protein, ILT5v1 and ILT5v2 based on their respective sequences [27]. Later, Bashirova and colleagues described six different “allotypes” of LILRB3 in the European population. Allotypes 2, 3, 4 and 6 share the same phylogenetic root and correspond to ILT5v1 while allotypes 1 and 5 corresponds to ILT5v2 [26]. These differences may carry the affinity of each variant for a specific ligand. Hence in 2016, Hofer and colleagues investigated LILRB3 variants binding capacity and determined that ILT5v2 bound complement factor C4d and class I HLA unlike ILT5v1 [28] C4d molecules binding to LILRB3 in activated monocytes inhibited TNF-α and IL-6 secretion in a dose dependant manner. Given the major role of complement activation in ABMR [29], the potential interaction of recipient-derived monocyte LILRB3 and C4d in transplant rejection context has yet to be determined.

The advent of *in silico* tools for modelling protein interactions such as HOMology modeling of COmplex Structure (HOMCOS) [30], allows to speculate that LILRB3 may interact with the heavy chain of antibodies (Fab heavy chain). The ability of LILRB3 to link a DSA Fab, while Fc fragment is bound to another immune cell, is an interesting process to investigate. This interaction could be sufficient to create an immunological synapse between two cells and to inhibit LILR specific pathways (including MAPK, PI3K/Akt or Jak/STAT) leading the monocytes to an anti-inflammatory phenotype. Imbalance of this interaction could switch the phenotype and favor a pro-inflammatory state therefore inducing donor cells destruction.

A large number of proteins have since been reported as potential ligands for LILRB3 including apolipoprotein-E (APOE) [31] and angiopoietin-like (ANGPTL). ANGPTL is involved in diabetes mellitus, in which it promotes the development of adipose tissue, chronic inflammation and systemic insulin resistance in response to T-cell and macrophages activation [32]. In addition, ANGPTL increases expression of TGF-ß and macrophage recruitment, leading to the development and progression of fibrosis in chronic kidney disease [33]. More recently, Galectin 4 and 7 were shown to bind and activate LILRB3 on the surface of myeloid cells and induce an immunosuppressive phenotype [21]. It’s a safe bet that the future will bring the discovery of other LILRB3 ligands, enriching LILRB3 with new functions, not only in the allograft context.

### LILRB3 as a Potential Non-Self Class I HLA Discriminator

**FIGURE 1:**
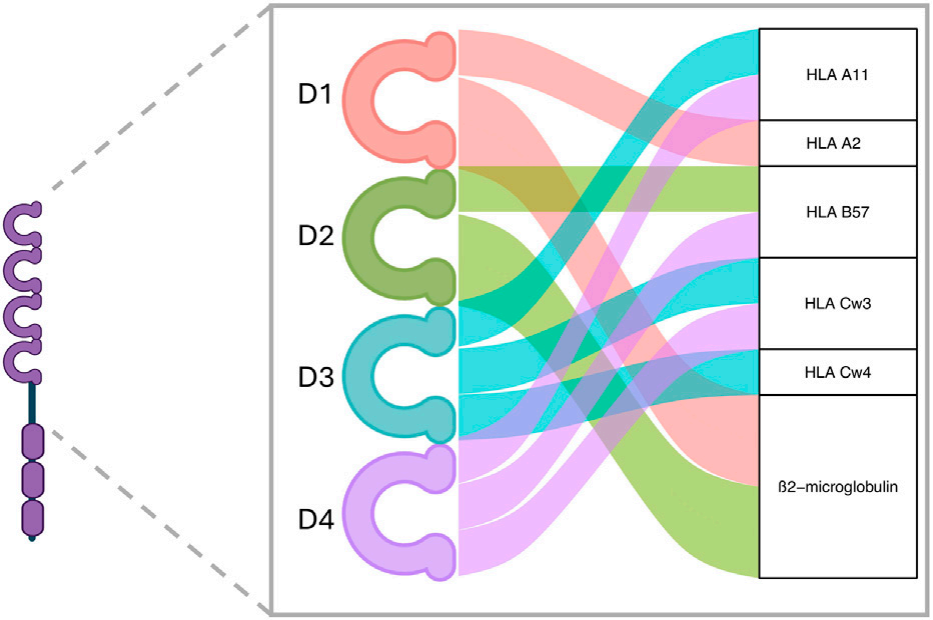
LILRB3’s predicted interaction domains with HLA molecules. Based on sequence alignment, several interaction sites with class I HLA are found on LILRB3 proteins. Domains 1 and 2 are predicted to mainly link the β2-microglobulin HLA class I invariant chain, whereas domains 3 and 4 are predicted to link several polymorphic HLA class I α-chains. Created with ggalluvial R package and BioRender.com.

To date, no LILRs have been described as potentially interacting with HLA class II molecules. However, with regard to LILRB3’s ability to bind HLA class I, the literature is contradictory. Indeed, the earliest reports claimed that LILRB3 was unable to bind HLA [34–37] whereas more recent reports [23, 28] contradict this assertion. These discrepancies could be due to the earlier mentioned LILRB3 various allotypes. *In silico* predictions suggest several potential contact sites for LILRB3 and HLA class I molecules interaction. Of the 4 immunoglobulin domains present in the extracellular part of LILRB3, domains 1 and 2 present binding sites to the invariant HLA class I ß2-microglobulin chain, as well as class I A2 molecule. In addition, domains 3 and 4 appear to have the most interaction sites with HLA I molecules A11, B57, Cw3, Cw4 (Figure 1). LILRB3 polymorphism, especially in domains 3 and 4, could be of utmost importance if balancing the affinity of a particular LILRB3 allotype to an allogeneic ligand, and thus determine the activation status of the recipient myeloid cells toward the donor cells.

### Sequence Homology With LILRA6

**FIGURE 2:**
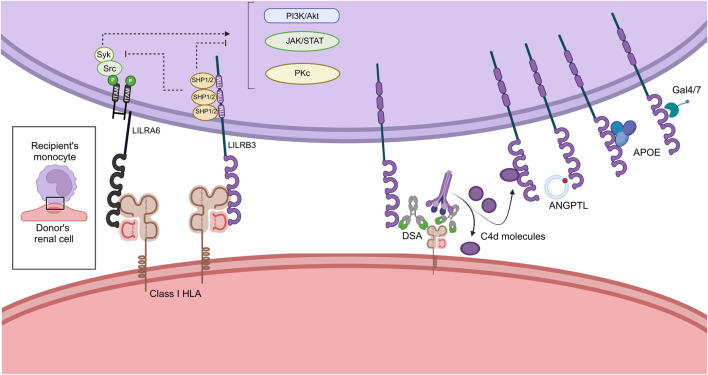
Immunologic synapse hypothesis between the donor’s renal cell and the recipient’s monocytic cell. ANGPTL, Angiopoietin-Like; APOE, Apolipoprotein-E; PI3K, Phosphoinositide 3 Kinase; DSA, Donor Specific Antibody; HLA, Human Leukocyte Antigen; ITAM, Immunoregulatory Tyrosin-based Activator Motifs; ITIM, Immunoregulatory Tyrosin-based Inhibitory Motifs; LILR, Leukocyte Immunoglobulin Like Receptor. Created with BioRender.com.

Intriguingly at the protein level, LILRB3 shows a strong homology of its extracellular domain with LILRA6, another polymorphic receptor with opposite functions [18, 26, 38]. As expected, this similarity is also found in the gene sequence encoding the two proteins, their homology making them difficult to distinguish either by transcriptomic approaches or using specific antibodies [26]. Unlike LILRB3, several copies of *LILRA6* gene can be present in the genome [38]. The homology between these two receptors implies that they bind the same proteins but induce opposite downstream signalization (Figure 2). In basal conditions, a balance exists between the LILRA6 positive signals and the LILRB3 negative signals. Upon strong binding to some LILRB3 allotypes, a disruption of this balance may happen, driving the cell into a specific phenotype. Overexpression of LILRB3 was mostly found on immunosuppressive cells in inflammatory contexts, supporting the hypothesis that LILRB3 induce anti-inflammatory signals in monocytes and leads to pro-resolutive phenotype [21]. With a greater copy number in addition to its high polymorphism, LILRA6 could outperform LILRB3 signals and lead to a pro-inflammatory phenotype. In the future, this sequence homology will have to be taken into account with the utmost vigilance, so that biological functions can be distinctly attributed to LILRB3 or LILRA6.

### Immunologic Synapse Hypothesis Between Recipient-Derived Macrophages and Donor Renal Cells During Graft Rejection

All known or putative interactions between LILRB3/A6 and their potential ligands of interest, leading to monocyte modulation, are summarized in Figure 2. In the context of kidney transplantation, the recipient-derived monocyte LILRB3 may interact with soluble C4d, directly with donor class I HLA but also with the Fc fragment of HLA-DSA bound to the surface of donor’s renal cells. Other ligands could also be secreted in the synapse for instance ANGPTL, APOE or Galectins, inducing downstream signalization. The ITIM sequences expressed in the LILRB intracellular domain recruits SHP1/2 phosphatases and induce inhibition of central signaling pathways such as PI3K/Akt, Jak/STAT and Pkc dependent pathways. Oppositely LILRA6 intracellular domain interact with ITAM leading to the recruitment of Syk and Src kinases. The recruitment of these proteins activates the same pathways and induce activation, differentiation and proliferation of the monocytes.

## Discussion

For many years, monocytes were regarded as mere second-knives, responding to non-specific danger signals and unable to trigger an allogeneic rejection on their own. New data derived from mouse models, have shown the potential of LILRs as key players in alloimmunity, suggesting their involvement in human allograft rejection. LILRs have been investigated in several context as modulators of the innate immune system activation. Yet their specific mechanism of action remains unclear. Recent founding proved the importance of LILRB3 in the cancer context, leading to the rapid development of anti-LILRB3 antagonist antibodies and chimeric-antigen receptor T-cells (CAR T-cells) to boost the anti-tumoral response [22]. In the context of transplant rejection, an opposite mechanism is expected, favoring a pro inflammatory phenotype and educating the recipient immune system against the donor’s cells. As LILRs high polymorphism drive their ligation capabilities, i.e., their ability to interact with donor cells, genotyping the recipient LILRB3 allotypes could improve our understanding of the monocytes-driven mechanisms of allorecognition. Interestingly, in African American transplant recipients, Sun and colleagues reported a potential association of the polymorphism in the *LILRB3* gene with long-term allograft outcomes, suggesting that this receptor is crucial in the allogeneic context. In fact, this polymorphism could be a genetic risk factor for graft outcome in this population [39]. Furthermore, a disruption in the balance between LILRB and LILRA expression on recipients’ monocytes in response to interaction with donor cells may be decisive in modulating the immunological synapse. This monocyte-driven allogeneic response is still at its infancy and many issues remain to be addressed. At first, it would be relevant to investigate whether LILRs expression is modulated on the surface of the recipient monocytes after allograft transplantation, and if the LILRA6/LILRB3 balance is tipped in the event of rejection. *In vitro*, what are the functional consequences for monocytes, if LILR expression is artificially increased or decreased? Can LILRs various allotypes sense the donor-recipient incompatibility? If so, it will be necessary to specify whether the trigger for monocyte activation is the presence of non-self HLA or rather the absence of self HLA (“missing-self”) as proposed for NK cells? Research teams will also need to focus on *in vitro* activation of human primary monocytes (a laboratory challenge given the “messiness” of these fragile cells) to better understand the cellular implications of LILR ligation to their various ligands. *In silico* binding predictions will need to be confirmed by experimental data to specify the range of molecules bound by each LILR.

Overall, these discoveries in innate immunity challenges the idea that ABMR rejection phenotype is solely caused by HLA and non-HLA DSA. It is likely that technological advances enabling cell phenotyping on an individual scale, will facilitate these investigations and provide more granularity in the involvement of innate immunity by causing or amplifying allogeneic response. Future developments include the opportunity of new therapeutic targets, whose need is clear to improve ABMR prognosis and long-term graft survival. Given that LILRs activate the PI3K/Akt and NFAT pathways, we can therefore speculate that calcineurin inhibitors or mTOR inhibitors might control monocyte activation in the context of allotransplantation. This field of LILRs activation pathways specific inhibitors is already a reality, with the Syk pathway inhibitor Fostamatinib FDA approved for the treatment of chronic immune thrombocytopenia.
